# Adipose-derived stem cell extracellular vesicles in diabetic microangiopathy: from pathological networks to clinical translation

**DOI:** 10.3389/fphar.2026.1970641

**Published:** 2026-10-26

**Authors:** Xinggui Zhu, Wenyan Yang, Yujie Ni, Yongyi Li, Jianming Wu, Yi Han, Qiaozhi Wang

**Affiliations:** 1 Key Laboratory of Medical Electrophysiology, Ministry of Education & Medical Electrophysiological Key Laboratory of Sichuan Province (Collaborative Innovation Center for Prevention of Cardiovascular Diseases), Institute of Cardiovascular Research, Southwest Medical University, Luzhou, China; 2 School of Basic Medical Sciences, Southwest Medical University, Luzhou, China; 3 Laboratory for Drug Discovery and Druggability Evaluation of Sichuan Province, Luzhou Key Laboratory of Activity Screening and Druggability Evaluation for Chinese Materia Medica, Southwest Medical University, Luzhou, Sichuan, China

**Keywords:** adipose-derived stem cells, diabetic microangiopathy, engineered extracellular vesicles, extracellular vesicles, pathological network remodeling, precision regenerative medicine

## Abstract

Diabetic microangiopathy (DMA) is a major cause of morbidity and mortality in diabetes. Its pathogenesis involves interconnected processes, including metabolic disturbance, oxidative stress, inflammation, immune dysregulation, endothelial dysfunction, mitochondrial abnormalities, and impaired tissue repair. This complexity suggests that therapeutic strategies targeting individual pathways may be insufficient to address the multidimensional pathological processes underlying DMA. Adipose-derived stem cell-derived extracellular vesicles (ADSC-EVs) have emerged as potential cell-free therapeutic candidates whose multimolecular cargo may modulate multiple disease-relevant processes. Preclinical evidence suggests that ADSC-EVs can influence oxidative stress, inflammation, angiogenesis, immune responses, and tissue repair, providing a potential basis for multitarget modulation of pathological microenvironments. This review critically evaluates organ-specific evidence for ADSC-EVs in diabetic retinopathy, diabetic kidney disease, diabetic peripheral neuropathy, and diabetic chronic wounds, with particular attention to evidence specificity, therapeutic mechanisms, delivery barriers, and translational limitations. We further summarize advances in EV engineering, including parent-cell and cargo engineering, surface modification, biomaterial integration, and stimuli-responsive delivery, while distinguishing ADSC-EV-specific findings from evidence extrapolated from the broader EV field. Finally, we discuss major barriers to clinical translation, including manufacturing standardization, potency assessment, biodistribution, organ-specific delivery, long-term safety, and regulatory evaluation. Future studies integrating disease stratification, multi-omics, rational engineering, and standardized manufacturing may help determine whether the multitarget effects of ADSC-EVs can be translated into reproducible and clinically meaningful therapies for DMA.

## 1 Introduction

Diabetic microangiopathy (DMA) includes chronic complications such as diabetic retinopathy (DR), diabetic kidney disease (DKD), diabetic peripheral neuropathy (DPN), and diabetic chronic wounds. Although they affect different tissues, these complications share metabolic stress, oxidative imbalance, AGE/RAGE-associated signaling, inflammation, and endothelial dysfunction (Brownlee, 2005; Forbes and Cooper, 2013; Schalkwijk and Stehouwer, 2020). These processes interact with organ-specific neurovascular injury and impaired tissue regeneration as the disease progresses. They can also reinforce one another: metabolic abnormalities can feed into inflammatory and vascular dysfunction, while impaired repair can allow tissue injury to persist even when some upstream insults are partly controlled. The local tissue environment adds another layer of complexity. The blood–retinal barrier in DR, the glomerular filtration barrier in DKD, and limited tissue accessibility in DPN can shape both disease manifestations and therapeutic access and responses (Cheung et al., 2010; Tuttle et al., 2014). Rather than viewing DR, DKD, DPN, and diabetic chronic wounds as separate microvascular disorders, we therefore consider them organ-specific manifestations that develop against a shared background of interacting pathological processes.

Such interconnected pathology provides a rationale for therapies that act on more than one disease process. Extracellular vesicles (EVs) are lipid-bilayer-delimited particles released by cells that participate in intercellular communication and can carry proteins, nucleic acids, lipids, and other bioactive components (Kalluri and LeBleu, 2020; Welsh et al., 2024). Among candidate EV sources, adipose-derived stem/stromal cell-derived EVs (ADSC-EVs) have received increasing attention as cell-free therapeutic candidates because their multimolecular cargo can influence inflammation, angiogenesis, cell survival, and tissue repair (Yang S. et al., 2024; Zhou et al., 2024). The ability to affect several processes, however, is not in itself evidence of pathological network remodeling. Here, this term refers to coordinated changes in interacting pathological modules and their feedback relationships, with the potential to shift a self-sustaining disease microenvironment toward a more reparative or homeostatic state. An important mechanistic question is therefore whether ADSC-EVs can alter these interactions at the network level or whether their reported effects largely reflect parallel actions on multiple molecular targets.

The literature on MSC-derived EVs in diabetes and its complications has expanded rapidly (Sun et al., 2022; Zhang et al., 2026), but several issues relevant to ADSC-EVs and diabetic microangiopathy remain unresolved. First, findings obtained directly from ADSC-EVs are often discussed together with those from EVs derived from other MSC sources, although EV composition and biological activity can vary with parent-cell source and culture context. Conclusions based specifically on ADSC-EV studies therefore need to be distinguished from those extrapolated from the broader MSC-EV literature. Second, studies and reviews have generally described the therapeutic effects of MSC-EVs in terms of individual cargos, signaling pathways, or pleiotropic biological effects. It remains unclear whether these changes collectively modify the interactions that sustain disease at the network level. Third, therapeutic optimization is not limited to modification of isolated EVs. It also includes upstream programming of EV-producing cells and engineering of delivery systems, and these approaches need to be considered together. Biological and methodological heterogeneity is another important issue. Differences in donor characteristics, culture conditions, and EV separation and characterization procedures continue to limit reproducibility and cross-study interpretation (Welsh et al., 2024). Variation in disease models, dosing regimens, and outcome measures further complicates comparisons among preclinical studies.

This review examines these issues across the path from disease mechanisms to therapeutic translation. We consider how pathological network remodeling differs from conventional pleiotropic or multitarget effects and assess the extent to which current evidence supports this concept in the context of ADSC-EVs. Particular attention is given to the source of the evidence, separating findings obtained directly with ADSC-EVs from those extrapolated from other MSC-EV sources or the broader EV field. We also examine therapeutic engineering at the levels of the parent cell, the EV itself, and the delivery system, together with the manufacturing and safety trade-offs associated with these approaches. Evidence in DR, DKD, DPN, and diabetic chronic wounds is then evaluated with respect to methodological quality, mechanistic support, reproducibility, and translational relevance. EV heterogeneity, biodistribution, safety, clinical evidence, and regulatory constraints are considered in relation to the remaining evidence gaps and priorities for developing reproducible, clinically meaningful ADSC-EV therapeutics.

## 2 Pathological network mechanisms of diabetic microangiopathy

Diabetic microangiopathy (DMA) involves disturbances across multiple pathological processes rather than disruption of a single pathway. Hyperglycemia-associated metabolic stress, oxidative imbalance, AGE/RAGE signaling, chronic inflammation, endothelial dysfunction, neurovascular injury, and impaired tissue repair are interconnected through molecular and cellular feedback mechanisms. Through these interactions, microvascular dysfunction, tissue hypoxia, cellular injury, and impaired regenerative capacity may reinforce one another and contribute to persistent and progressive tissue damage.

This interconnected view of DMA helps explain how these pathological processes contribute to disease progression and why targeting individual molecular pathways may have limited effects. However, an intervention that affects several targets does not necessarily alter the interactions that sustain the disease network. The distinction between such pleiotropic effects and pathological network remodeling is discussed in Section 2.4.

### 2.1 The metabolic–oxidative stress axis

Chronic hyperglycemia is a major metabolic factor in DMA progression. Sustained hyperglycemia increases mitochondrial ROS production and contributes to several pathways involved in hyperglycemic injury, including the polyol pathway, protein kinase C (PKC) signaling, AGE formation, and increased flux through the hexosamine pathway (Nishikawa et al., 2000; Brownlee, 2005). Diabetes can also impair endogenous antioxidant defenses, further disturbing the balance between ROS production and antioxidant capacity (Pitocco et al., 2013; Volpe et al., 2018).

Excess ROS can damage nucleic acids, lipids, proteins, and mitochondrial structures. They can also disrupt endothelial nitric oxide signaling, mitochondrial energy metabolism, and vascular homeostasis. Oxidative stress is therefore more than a downstream consequence of metabolic dysfunction. It is closely linked to inflammatory signaling, endothelial dysfunction, and progressive vascular injury and, in this way, connects metabolic abnormalities with other pathological processes in diabetic complications (Giacco and Brownlee, 2010).

Reducing oxidative stress has not consistently produced substantial clinical benefit in diabetic complications. ROS are not simply harmful by-products that can be removed without affecting other processes. They also participate in inflammatory and vascular signaling. Their effects depend partly on the tissue involved, since metabolic demands, cellular composition, and local microenvironments differ among organs.

This may help explain why reducing ROS alone is unlikely to address the full metabolic–oxidative disturbance in DMA. Metabolic stress and ROS are linked to mitochondrial dysfunction as well as inflammatory and vascular responses. ADSC-EVs have been reported to affect oxidative stress, inflammatory signaling, and tissue-repair processes, making them relevant to the study of multitarget intervention (Zhou et al., 2024). Even so, effects on several pathways should not be taken as evidence that the pathological network itself has been remodeled.

### 2.2 The inflammation–endothelial dysfunction axis

Chronic hyperglycemia promotes the accumulation of advanced glycation end products (AGEs), which engage RAGE and activate inflammatory pathways including NF-κB (Schmidt et al., 2000; Ott et al., 2014). Adhesion molecules such as ICAM-1 are also upregulated, facilitating leukocyte adhesion and vascular injury, as shown in experimental diabetic retinopathy (Joussen et al., 2004). Alongside these changes, chronic inflammation and oxidative stress disturb endothelial homeostasis, reduce nitric oxide bioavailability, alter vascular permeability and vasomotor responses, and impair microvascular perfusion and regeneration.

Inflammation and endothelial dysfunction are closely linked during DMA progression. AGE/RAGE–NF-κB signaling can promote both inflammatory activation and endothelial injury, while damaged vessels can maintain a local inflammatory environment. These changes may persist in vascular and immune cells rather than behaving as a short-lived inflammatory response. This makes inflammation difficult to separate from the accompanying vascular dysfunction. Whether targeting both processes is more effective than targeting individual pathways, however, remains uncertain.

Improved glycemic control does not always reverse vascular and inflammatory abnormalities that have already developed. This persistence, commonly termed metabolic memory, has been linked to epigenetic alterations, oxidative stress, AGE/RAGE signaling, and mitochondrial abnormalities (Reddy and Natarajan, 2011; Ceriello, 2012).

AGE/RAGE signaling should therefore not be viewed in isolation. It operates alongside oxidative stress, endothelial dysfunction, immune dysregulation, and metabolic abnormalities, with each process potentially influencing the others and sustaining microvascular injury.

Once these interactions are established, changing a single inflammatory mediator may not be enough to reverse microvascular dysfunction. EV-based and other multitarget approaches may affect several parts of this inflammatory–vascular axis. That would demonstrate activity across multiple processes, but not necessarily durable remodeling of the pathological network. Direct mechanistic evidence would still be needed to support the latter conclusion.

### 2.3 The neurovascular–repair imbalance axis

Diabetic microvascular complications, particularly diabetic retinopathy (DR), involve more than vascular injury alone. Dysfunction of the neurovascular unit (NVU) is also involved in disease progression (Stitt et al., 2016; Simó et al., 2018). Neural injury and microvascular abnormalities are closely linked, although their relationship differs among tissues and it remains unclear to what extent they form self-sustaining bidirectional feedback loops.

This relationship is also relevant to diabetic peripheral neuropathy (DPN). Schwann cells, axons, and the local microvasculature interact within the same tissue environment, making it difficult to view neural and vascular abnormalities as independent processes (Gonçalves et al., 2017). Reduced microvascular support may contribute to neural dysfunction, while changes in nerve-associated cellular interactions may further disturb the local microenvironment. Neural–vascular interactions have also been described in DR, suggesting that neurovascular dysfunction is relevant to more than one diabetic microvascular complication (Stitt et al., 2016).

These interactions are also relevant when considering treatment. Restoring vascular support alone may not adequately address established neural injury. Approaches that support both vascular function and neural repair may therefore be useful in complications with neurovascular dysfunction, although whether they are more effective than targeting either component alone remains to be demonstrated.

### 2.4 Integration of pathological networks: From pleiotropic effects to pathological network remodeling

AGE/RAGE signaling, oxidative stress, inflammation, and microvascular dysfunction are often discussed separately, although they are closely linked in diabetic microvascular injury (Koulis et al., 2015). AGE/RAGE activation can increase ROS production, which can enhance NF-κB-mediated inflammatory signaling. Persistent oxidative and inflammatory stress can then aggravate mitochondrial and endothelial dysfunction. These processes may reinforce one another and help sustain the diabetic microvascular environment. Their effects also vary across tissues because cellular composition and local microenvironments differ among DMA manifestations.

In this review, we distinguish pathological network remodeling from pleiotropic or multitarget effects. We use pleiotropic or multitarget effects to describe changes in multiple molecular targets, signaling pathways, or cellular phenotypes. Pathological network remodeling refers to coordinated changes in interacting pathological modules and, importantly, in the feedback relationships that sustain them, resulting in a measurable shift away from a self-reinforcing disease state. This distinction matters when interpreting experimental findings. For example, simultaneous reductions in oxidative stress and inflammation together with increased angiogenic or regenerative markers demonstrate multitarget activity, but these findings alone do not establish network remodeling. Evidence for network remodeling would need to show that these changes are functionally connected and lead to sustained modification of disease-associated feedback loops.

ADSC-EVs contain multiple bioactive components and have been reported to affect several disease-related processes, making network-level regulation plausible. Current evidence, however, does not demonstrate that ADSC-EVs remodel pathological networks. Most studies focus on selected cargos, signaling pathways, or phenotypic endpoints and do not test causal relationships among different pathological modules. We therefore consider pathological network remodeling a testable framework for interpreting the multitarget effects of ADSC-EVs rather than an established mechanism. Direct evidence for coordinated and durable changes in disease-sustaining networks remains limited.

Multi-omics studies are beginning to reveal coordinated molecular alterations in individual diabetic complications, particularly DKD, and broader integrative analyses have identified both shared and tissue-specific molecular features across diabetic microvascular complications (Liu et al., 2021; Li et al., 2024; Singh et al., 2025; Grzybowska-Adamowicz and Zmysłowska, 2026). EVs also mediate intercellular communication through the transfer of proteins, nucleic acids, lipids, and other bioactive molecules (Raposo and Stoorvogel, 2013; van Niel et al., 2018). Together, these approaches may help identify coordinated changes across pathological modules. They cannot, however, establish network remodeling on their own. Causal perturbation and longitudinal functional studies are needed to determine whether the observed changes alter disease-sustaining interactions or simply occur in parallel.

Figure 1 brings these concepts together by linking the metabolic–oxidative stress, inflammation–endothelial dysfunction, and neurovascular–repair imbalance axes. It also distinguishes multitarget activity from the additional evidence needed to demonstrate pathological network remodeling.

**FIGURE 1:**
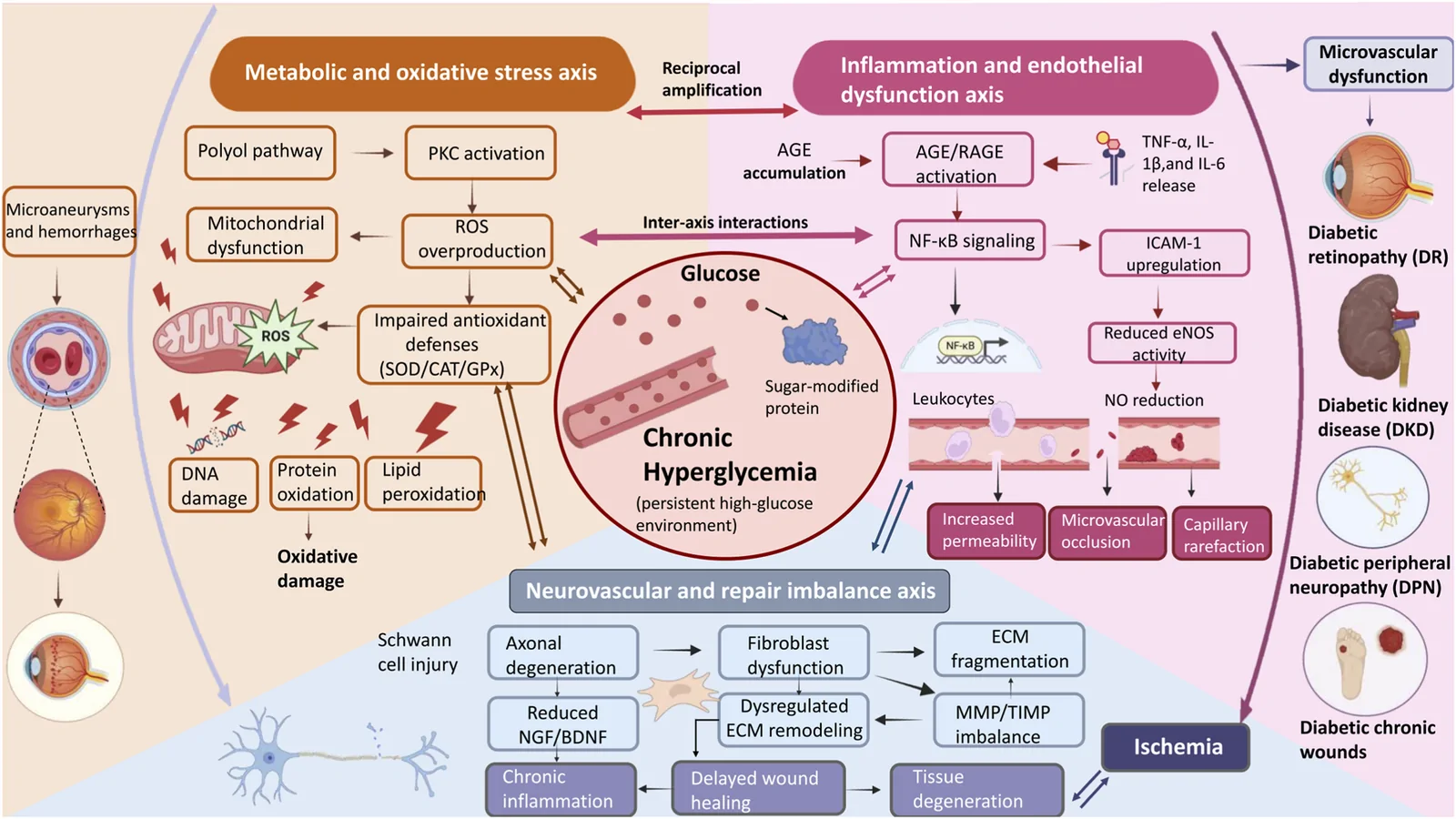
Systems-level framework of the interconnected pathological network in diabetic microangiopathy. Chronic hyperglycemia contributes to three interacting pathological axes: (1) the metabolic–oxidative stress axis, including the polyol pathway, PKC activation, mitochondrial dysfunction, and ROS overproduction; (2) the inflammation–endothelial dysfunction axis, involving AGE/RAGE signaling, NF-κB activation, cytokine release, eNOS inhibition, and microvascular abnormalities; and (3) the neurovascular–repair imbalance axis, involving Schwann cell injury, axonal degeneration, reduced neurotrophic support, and abnormal ECM remodeling. Interactions among these processes may amplify microvascular injury and contribute to microcirculatory dysfunction and organ-specific tissue damage in diabetic retinopathy (DR), diabetic kidney disease (DKD), diabetic peripheral neuropathy (DPN), and diabetic chronic wounds. The figure conceptually integrates interacting pathological processes rather than providing evidence that all depicted relationships are experimentally established causal feedback loops. Created in BioRender. Yang, W. (2026) https://BioRender.com/cpgcfzs.

## 3 Biological rationale and evidence basis for ADSC-EVs

Mesenchymal stem/stromal cells (MSCs), including adipose-derived stem/stromal cells (ADSCs), have been widely studied in regenerative medicine because of their immunomodulatory, pro-angiogenic, and tissue-reparative activities (Caplan, 2017; Pittenger et al., 2019). Their use as direct cell therapies, however, is limited by challenges such as poor cell persistence and engraftment, variable therapeutic efficacy, and potential biosafety concerns. Moreover, the therapeutic effects of MSCs do not appear to depend entirely on long-term cellular engraftment. Paracrine mechanisms contribute to at least part of their activity (Gnecchi et al., 2008; Vizoso et al., 2017).

Extracellular vesicles (EVs) are one component of this paracrine activity and have therefore been investigated as cell-free therapeutic candidates. They can transfer bioactive molecules between cells and reproduce some of the paracrine effects of their parent cells without transplantation of viable cells (Phinney and Pittenger, 2017). The biological properties of EVs, however, vary with their cellular origin and production conditions. For this reason, we distinguish evidence obtained specifically from ADSC-EVs from properties reported more generally for MSC-EVs or EVs from other cellular sources.

### 3.1 ADSC-EVs as multimolecular delivery platforms

ADSC-EVs share the basic structural and functional properties of EVs from other cellular sources. Their lipid bilayer can protect bioactive cargo during transfer to recipient cells, which occurs through several interaction and uptake mechanisms (Kalluri and LeBleu, 2020). EV cargo can include regulatory RNAs, proteins, lipids, and metabolites, each of which may affect recipient-cell functions (Kalluri and LeBleu, 2020; O'Brien et al., 2020). The resulting biological activity may reflect the combined effects of several cargos rather than the action of a single component. These features are common to EV biology and are not specific advantages of ADSC-EVs.

This capacity to carry multiple bioactive components is one reason EVs have been considered as multitarget therapeutic platforms (El Andaloussi et al., 2013; Kalluri and LeBleu, 2020). ADSC-EVs could therefore influence several processes relevant to DMA, but the extent to which these effects are specific to ADSC-EVs requires direct evidence. Moreover, as discussed in Section 2.4, effects on multiple targets alone are not sufficient to establish pathological network remodeling.

### 3.2 Molecular composition and the basis for multitarget regulation

EVs contain heterogeneous mixtures of RNAs, proteins, lipids, and other molecular components. Much of the evidence describing this cargo composition, however, comes from heterogeneous EV populations or MSC-EVs derived from sources other than adipose tissue (Théry et al., 2006; Ferguson and Nguyen, 2016; Kowal et al., 2016). These studies show that EV cargo is molecularly diverse, but they do not define a molecular profile specific to ADSC-EVs.

ADSC-EVs also show considerable variation in cargo composition, depending in part on parental-cell characteristics and culture conditions. This variability needs to be considered when assigning therapeutic mechanisms to ADSC-EVs, as mechanisms identified in other MSC-EV populations may not necessarily apply to them. Figure 2 summarizes the reported molecular diversity of ADSC-EVs and how these cargos may relate to different disease-relevant biological processes.

**FIGURE 2:**
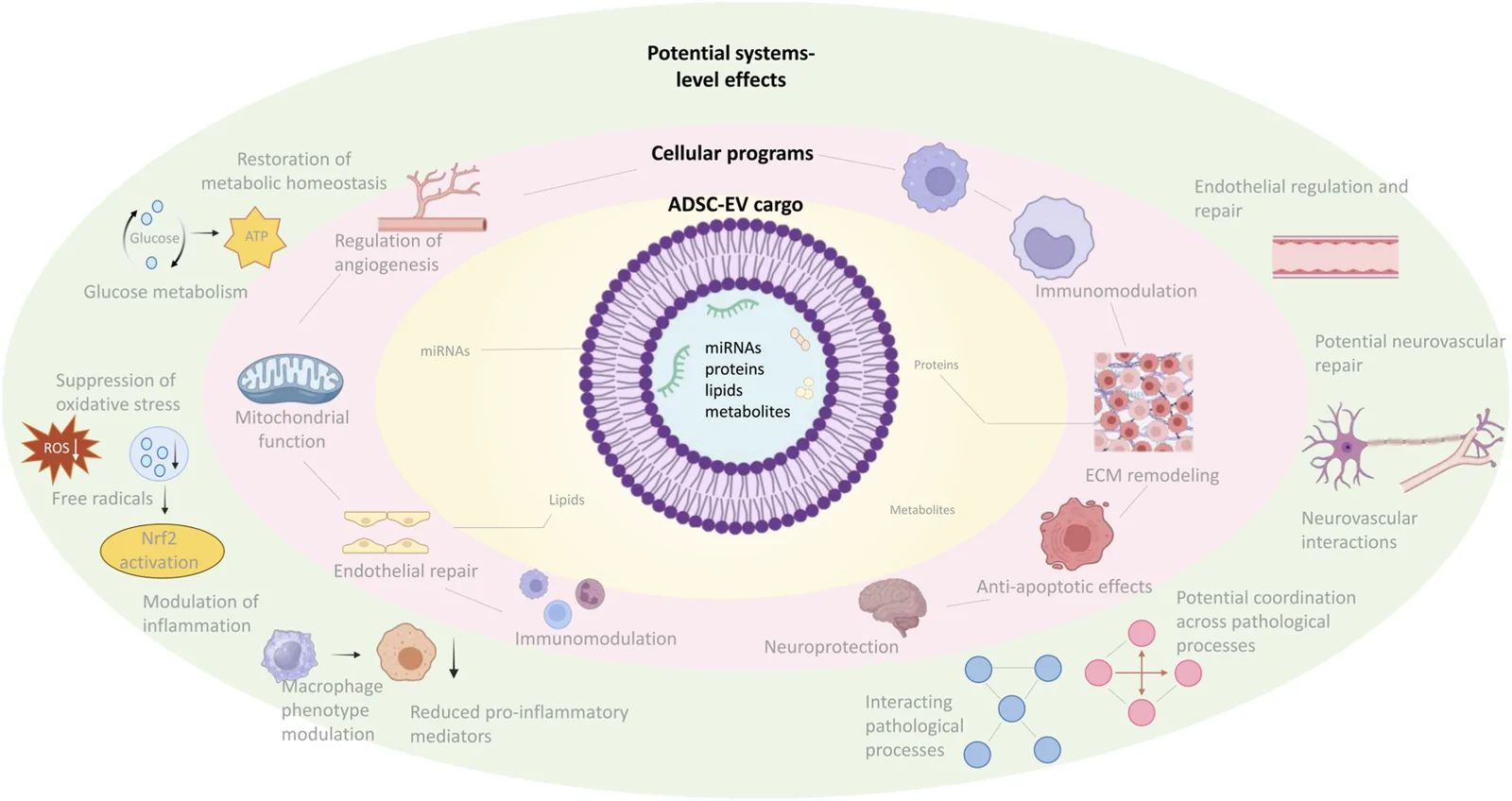
Molecular cargo diversity and proposed multitarget biological effects of ADSC-derived extracellular vesicles. ADSC-EVs contain heterogeneous molecular cargo, including regulatory RNAs, proteins, lipids, and metabolites. Available experimental studies suggest that ADSC-EVs can influence multiple disease-relevant biological processes, including oxidative stress, inflammatory responses, angiogenesis, endothelial function, extracellular matrix remodeling, cell survival, and neural repair. These effects provide a biological basis for considering ADSC-EVs as multimolecular and multitarget therapeutic candidates. However, individual cargos and biological effects vary across experimental conditions, and evidence derived from other MSC-EV sources should not be interpreted as ADSC-EV-specific evidence. Importantly, simultaneous modulation of multiple processes does not by itself demonstrate pathological network remodeling; establishing network-level remodeling will require evidence that these effects are functionally interconnected and produce sustained changes in disease-associated feedback relationships. Created in BioRender. Yang, W. (2026) https://BioRender.com/k7r449e.

The broader MSC-EV literature provides examples of how particular cargos can affect specific biological responses. Exosomes derived from miR-126-overexpressing MSCs, for example, promoted angiogenic activity through PIK3R2/PI3K/Akt signaling (Zhang et al., 2021). This finding demonstrates a mechanism by which an EV-associated cargo can exert biological activity. It does not show that the same cargo is endogenously enriched or has comparable functional importance in ADSC-EVs.

It also remains unclear how much individual cargos contribute to the overall therapeutic activity of EVs (Tkach and Théry, 2016; Pegtel and Gould, 2019). In ADSC-EVs, several molecular components may contribute to biological activity, with their effects depending on the recipient cell and disease context. Cargo diversity can therefore support multitarget regulation without implying that a pathological network has been remodeled. Demonstrating network remodeling will require more than molecular profiling or changes in several pathways; causal perturbation and functional studies are needed to determine whether disease-relevant pathways are altered in a coordinated and interconnected manner.

### 3.3 ADSC-EVs in the context of other MSC-derived EVs

ADSC-EVs have received increasing attention as cell-free therapeutic candidates, but current evidence does not establish their general superiority over EVs from other MSC sources. MSCs derived from adipose tissue, bone marrow, and umbilical cord differ in their biological characteristics, proliferative capacity, donor-related variables, and tissue-acquisition requirements, and these differences may also affect their EV products. No single MSC-EV source can therefore be considered optimal for all applications. Source selection needs to account for the intended therapeutic use as well as biological activity, manufacturing feasibility, and product consistency. For ADSC-EVs, their translational value may lie less in demonstrated biological superiority than in the relatively high cellular yield from adipose tissue and the feasibility of expanding these cells for EV production.

Parental-cell characteristics alone, however, do not determine the properties of an EV product. EV yield, molecular composition, and biological activity can also vary with culture and manufacturing conditions. Meaningful comparisons among ADSC-EVs, BM-MSC-EVs, and UC-MSC-EVs therefore require standardized production and characterization methods, together with comparable dosing and functional assays.

BM-MSCs are among the most extensively studied MSC sources in regenerative medicine (Squillaro et al., 2016). However, their collection requires invasive bone marrow harvesting. UC-MSCs, in contrast, can be obtained from postpartum tissues without additional invasive tissue harvesting. Their favorable proliferative and immunomodulatory characteristics also make them attractive for allogeneic applications (Batsali et al., 2013; Wegmeyer et al., 2013).

Adipose tissue is abundant and can yield relatively large numbers of stromal/stem cells. Its collection is nevertheless invasive, usually requiring liposuction or surgical adipose-tissue harvesting. ADSCs should therefore not be described as intrinsically more accessible than UC-MSCs. Their relatively high cell yield and expansion potential may still be useful for large-scale cell expansion and EV production. Table 1 summarizes the main source-related characteristics, practical advantages, and limitations of BM-MSCs, UC-MSCs, and ADSCs relevant to EV production.

**TABLE 1:** Comparison of major source-related characteristics relevant to EV production among bone marrow-, umbilical cord-, and adipose-derived MSCs.

MSC source	Major advantages relevant to EV production	Major limitations	References
Bone marrow-derived MSCs (BM-MSCs)	Extensively investigated MSC source with substantial experimental and clinical experience	Invasive tissue harvesting; donor age may affect parental-cell characteristics	(Baker et al., 2015; Squillaro et al., 2016)
Umbilical cord-derived MSCs (UC-MSCs)	*Postpartum* acquisition without an additional invasive harvesting procedure; favorable proliferative characteristics; potential suitability for allogeneic applications	Donor heterogeneity; variability associated with tissue processing and manufacturing procedures	(Wegmeyer et al., 2013; Mebarki et al., 2021)
Adipose-derived stem/stromal cells (ADSCs)	Abundant tissue source; relatively high cell yield and expansion potential; potential manufacturing scalability	Invasive tissue harvesting; donor metabolic status may influence parental-cell characteristics, with potential implications for EV-product variability	(Bunnell, 2021; Fang et al., 2022)

Donor and parental-cell factors may introduce additional variability into ADSC-EV production. Obesity-associated changes in the adipose microenvironment, for example, have been linked to increased ER stress and senescence in ADSCs, suggesting that donor metabolic status may affect the biological state of EV-producing cells (Fang et al., 2022). Whether these changes lead to reproducible differences in ADSC-EV composition, potency, or therapeutic efficacy remains unclear. Clinical development will therefore require defined donor-selection criteria as well as standardized manufacturing and potency assessment.

ADSC-EVs are thus a practically relevant MSC-EV source, particularly because of parental-cell yield, expansion capacity, and potential manufacturing scalability. These advantages are context-dependent and should not be taken as evidence of biological superiority over EVs from other MSC sources. Direct comparisons under standardized EV production, characterization, dosing, and functional-assay conditions are needed to determine whether specific MSC-EV sources offer advantages for particular applications in diabetic microangiopathy.

## 4 Engineering strategies for ADSC-EVs: from parent-cell engineering to precision delivery

Preclinical studies have reported therapeutic effects of ADSC-EVs in selected diabetic complications, but several features of native EV preparations may limit their therapeutic use. Production yield can be low, EVs may be rapidly cleared after administration, and their tissue targeting and retention are often limited. Control over cargo loading and delivery also remains challenging (Wang et al., 2023; Yang C. et al., 2024). Different engineering approaches have been investigated to address these problems, with the aim of improving EV production, molecular properties, targeting, retention, or delivery (Rhim et al., 2023; Wang et al., 2023). We consider these approaches at three levels. Parent-cell programming modifies ADSCs before EV production to influence the EVs they release. EV-level modification acts directly on EV cargo or surface properties, whereas delivery engineering uses biomaterials or other delivery systems to improve local retention, controlled release, or responsive delivery (Rhim et al., 2023; Yang C. et al., 2024). These approaches can be used separately or combined, depending on the therapeutic objective, rather than following a fixed sequence. Each additional modification, however, can make the final product more difficult to manufacture and characterize consistently and may introduce further biosafety, scalability, and regulatory challenges. Figure 3 provides an overview of these strategies and their potential therapeutic implications.

**FIGURE 3:**
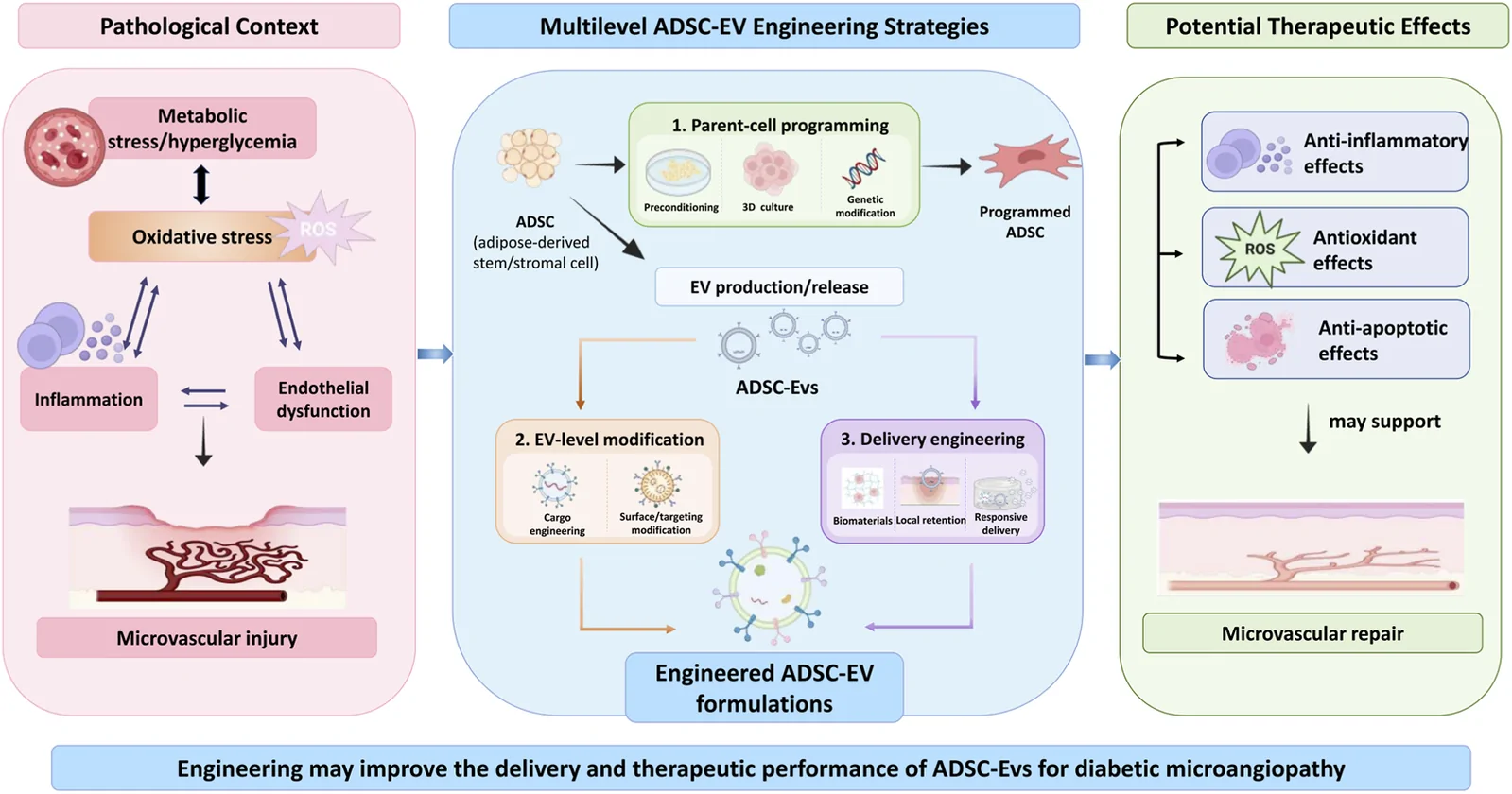
Multilevel engineering strategies for ADSC-EV-based therapy in diabetic microangiopathy. ADSC-EV engineering can be implemented at multiple complementary levels, including parent-cell programming through preconditioning, three-dimensional culture, or genetic modification; EV-level modification through cargo engineering or surface/targeting modification; and delivery engineering using biomaterials, local-retention approaches, or responsive delivery systems. These strategies may improve EV production, molecular or surface properties, targeting, retention, and delivery performance, thereby potentially enhancing anti-inflammatory, antioxidant, and anti-apoptotic effects and supporting microvascular repair. These engineering approaches are complementary and may be applied independently or in combination rather than as obligatory sequential steps. Created in BioRender. Yang, W. (2026) https://BioRender.com/3rzoxhr.

### 4.1 Parent-cell engineering to optimize EV production and function

EV properties can be modified before isolation by changing the conditions under which parental ADSCs produce them. Culture architecture, microenvironmental conditions, and donor-cell gene expression can all affect EV yield and molecular cargo (Rhim et al., 2023). Three-dimensional (3D) culture, hypoxic preconditioning, and genetic modification of donor ADSCs are the main approaches used for this purpose. Unlike downstream EV engineering, these methods act on the cells that produce the vesicles.

Three-dimensional culture changes cell–cell interactions and the local cellular environment and, in turn, can alter ADSC secretory activity. Studies comparing 3D and conventional two-dimensional culture have reported differences in both ADSC-EV production and biological properties (Li Y. et al., 2025; Jiang et al., 2026). The effects are not straightforward, however, and depend on how the system is designed. In the study by Jiang et al., exosomes from 3D-cultured ADSCs were incorporated into a hybrid scaffold for diabetic wound treatment. The *in vivo* effect therefore reflects both the EV preparation and the delivery scaffold and cannot be assigned to 3D culture alone. Wang et al. more recently developed a self-feeder layer 3D (SFL-3D) system to generate three-dimensional adipose stem cells (tdASCs) (Wang et al., 2026). SFL-3D culture increased EV production, and EVs produced by these cells (tdASC-EVs) showed greater therapeutic activity. In a diabetic wound model, tdASC-EVs alleviated high-glucose-induced microvascular endothelial senescence through the PI3K/AKT/mTOR/4E-BP1 axis. The authors also tested tdASC-EVs in a diabetic porcine wound model, where they accelerated wound closure, increased neovascularization, and reduced scar formation more effectively than conventional ADSC-EVs (Wang et al., 2026).

ADSCs can also be preconditioned before EV collection. Hypoxic culture has been shown to alter ADSC-EV cargo profiles and biological properties, and hypoxia-preconditioned ADSC-EVs have been associated with enhanced angiogenic and regenerative activities (Han et al., 2018; Han et al., 2019). Hypoxia does not, however, consistently increase the number of EVs produced. Geßner et al. reported changes in EV protein cargo without a significant increase in EV number (Geßner et al., 2021). The response to hypoxic preconditioning therefore varies across experimental conditions. An improvement in EV activity under one protocol should not be assumed to apply to other ADSC-EV preparations.

A more targeted option is to modify gene expression in the parental ADSCs. For example, exosomes derived from mmu_circ_0000250-modified ADSCs promoted wound healing in diabetic mice through a miR-128-3p/SIRT1-associated autophagy mechanism (Shi et al., 2020). This work shows that genetic manipulation of ADSCs can change the therapeutic properties of the vesicles they release in a diabetes-related wound model. It also adds another layer of complexity to production, with implications for reproducibility, product heterogeneity, biosafety, and large-scale manufacturing. Parent-cell engineering can complement downstream cargo and surface modification, but higher EV yield or greater experimental efficacy alone does not determine its translational value. The resulting EV product must also be consistent, potent, and safe.

### 4.2 Cargo engineering: enhancing therapeutic functionality

EV cargo can be changed before or after vesicle isolation. Modifying the parental cells, as discussed in Section 4.1, alters the cargo during EV production. A different option is to load selected nucleic acids, proteins, or small-molecule agents into EVs after they have been isolated. This approach allows more direct control over the cargo being introduced. Most of the available evidence, however, comes from the broader EV field rather than from ADSC-EVs studied specifically in diabetic microangiopathy.

Co-incubation, electroporation, sonication, and freeze–thaw procedures have all been used for post-isolation loading (Song et al., 2024). None is without limitations. Electroporation transiently permeabilizes the EV membrane and can facilitate cargo entry, whereas sonication may increase cargo incorporation but can also alter EV structure or biological activity. The amount of cargo loaded is therefore only one part of the assessment. Any gain in loading efficiency needs to be considered together with possible changes in EV integrity and function.

In diabetes and its complications, engineered EVs carrying selected therapeutic molecules have been studied for effects on inflammation, angiogenesis, and tissue repair (Liu S. Y. et al., 2025). The evidence is much less developed for post-isolation engineering of ADSC-EVs in diabetic microangiopathy. In particular, there is still limited evidence that these engineered preparations provide greater therapeutic benefit than unmodified ADSC-EVs. Studies addressing this question need to look beyond loading efficiency and determine whether the process can be reproduced, whether the loaded cargo remains stable, and whether the resulting EVs retain their identity, integrity, potency, and safety.

### 4.3 Surface engineering: improving lesion-specific targeting

Modifying the EV surface can change how vesicles interact with cells and tissues and may help direct them toward specific disease sites. Methods developed in the broader EV field include adding targeting peptides, aptamers, or other membrane-associated ligands (Kang et al., 2020). Some of these approaches have been tested in diabetes-related models. Endothelial-targeting aptamer-functionalized exosomes, for example, promoted angiogenesis and wound healing in a type 1 diabetic mouse model. Targeted engineered EVs have also been studied for their ability to ameliorate inner blood-retinal barrier dysfunction in experimental diabetic retinopathy (Fan et al., 2025; Zhao et al., 2025).

Neither example, however, provides direct evidence for surface-engineered ADSC-EVs, as these studies largely used EVs from other cellular sources. Evidence that surface modification improves lesion-specific targeting of ADSC-EVs in diabetic microangiopathy is still limited. We therefore view findings from the broader engineered-EV literature as proof of principle rather than evidence that the same benefits have been established for ADSC-EVs.

Surface modification can also affect more than targeting. Changes to the EV membrane may alter physicochemical properties, biodistribution, biological activity, and potentially immunological behavior. These effects need to be considered alongside any improvement in tissue targeting. For clinical use, modification methods will also need to be reproducible and scalable, with EV integrity, potency, biodistribution, targeting specificity, and safety assessed throughout development.

### 4.4 Local delivery systems: prolonging therapeutic retention

One way to keep EVs at the treatment site for longer is to incorporate them into hydrogels. GelMA, alginate, hyaluronic acid, and other biomaterials have been used for this purpose in diabetic complications (Long et al., 2024; Cong et al., 2025; Ju et al., 2025; Tian et al., 2025; Zhang et al., 2025). In diabetic wound models, EV-loaded hydrogels have produced better outcomes than free EVs or relevant control formulations. The hydrogel can limit early EV loss or degradation while also providing a local environment that supports repair. The same idea has been tested beyond diabetic wounds. Bakhtiari et al., for example, used an in situ-forming PEG hydrogel for sustained MSC-EV delivery in DKD (Bakhtiari et al., 2025). These studies are not directly comparable, however, because they use different EV sources, engineering methods, and hydrogel formulations, and only some specifically examine ADSC-derived EVs.

The hydrogel is not simply an inert carrier. Its polymer composition and crosslinking influence how EVs are released, while interactions between the EVs and the material can affect EV stability and biological activity. Conditions within the surrounding tissue may alter these properties further. In addition, the material itself may contribute to tissue repair. Better outcomes with an EV-loaded hydrogel therefore cannot automatically be attributed to improved EV retention. Appropriate controls are needed to determine what comes from the EVs and what comes from the delivery material. Hydrogel composition, EV loading, release behavior, and product quality will also need to be controlled more consistently if these systems are to move toward clinical use.

### 4.5 Microneedle-based delivery: improving local tissue accessibility

Microneedles offer a minimally invasive way to deliver therapeutic agents across the stratum corneum into superficial tissues. Several studies have incorporated EVs into microneedles for diabetic wound treatment, often combining EV delivery with other functions of the material. Exosome-loaded metal–organic framework microneedles have been used for local delivery to diabetic wounds (Xiang et al., 2025). In another approach, conductive microneedles containing polyphenol-engineered vesicles combined vesicle delivery with electrical stimulation (Liu D. et al., 2025). Dissolving microneedles containing plant-derived vesicles have also been tested in diabetic wounds (Ye et al., 2025).

These studies show what microneedle-based vesicle delivery can achieve, but they do not provide direct evidence for ADSC-EVs. The platforms use different vesicle sources and materials, and some include additional therapeutic components. This also makes their effects difficult to assign to EV delivery alone, since the microneedle itself or the accompanying intervention may contribute to the outcome. The anatomical reach of conventional transdermal microneedles is another constraint. They are mainly suited to superficial tissues and have less direct relevance to internal complications such as diabetic kidney disease and diabetic retinopathy. It therefore remains unclear whether microneedles provide a reproducible advantage for ADSC-EV delivery, and their usefulness is likely to depend on the disease being treated.

### 4.6 Stimuli-responsive platforms and manufacturing considerations

Some delivery systems are designed to respond to conditions within diseased tissue and release their therapeutic cargo accordingly. Ma et al., for example, developed a ROS-responsive hydrogel containing M2 macrophage-derived nanovesicles that released the vesicles in response to ROS in a diabetic wound model (Ma et al., 2025). This type of system offers a way to link vesicle release to the local pathological environment and potentially improve control over when and where delivery occurs. However, the vesicles used in this study were not derived from ADSCs, and it remains unclear whether the same strategy would provide similar benefits for ADSC-EVs. Adding responsive materials and other components also makes the final product more complex, which can complicate characterization, reproducibility, stability, and safety assessment.

Scaling up the production of engineered EVs presents another challenge. Microfluidic technologies, scalable culture platforms, and other production methods have been explored to increase EV yield and improve manufacturing control (Hassanzadeh-Barforoushi et al., 2025; Huang et al., 2025). Microbead-based culture has also been used to increase the production of human MSC-derived EVs (Tan et al., 2025). However, producing more EVs is only part of the problem. For clinical use, EV products must also maintain consistent characteristics and potency from batch to batch. This makes standardized characterization, potency testing, and quality control necessary throughout the production process.

### 4.7 Translational trade-offs and design considerations

Improving the biological activity of EVs is only useful if enough of the administered product reaches the tissue of interest. After systemic administration, EVs are distributed unevenly across the body, which can reduce exposure at the intended target site (Kang et al., 2021). Good performance *in vitro* therefore does not necessarily predict sufficient therapeutic exposure *in vivo*. Distribution may also depend on the condition of the recipient. Experimental systemic inflammation, for example, can alter EV clearance and tissue distribution (Pavlova et al., 2026). Targeting strategies, controlled-release systems, and approaches that increase local retention may improve EV exposure, but it is still unclear whether these changes will make ADSC-EV therapy more practical for clinical use.

The same engineering approach is unlikely to work equally well for every DMA manifestation. Diabetic chronic wounds are accessible to local treatment and may benefit from systems that keep EVs at the wound site. The retina and kidney present different anatomical and physiological barriers and therefore require different solutions. Even an effective delivery system must also be practical to manufacture and evaluate. Scale-up, consistent quality control, biosafety, and regulatory requirements remain important parts of clinical development, as discussed further in Section 6. Table 2 summarizes the main engineering strategies, their potential advantages and limitations, the boundaries of the available evidence, and their translational considerations. The choice of EV engineering strategy should therefore reflect the organ being treated and the specific demands of the indication.

**TABLE 2:** Major engineering strategies for ADSC-EV therapeutic development and their translational considerations.

Engineering strategy	Representative approaches	Potential advantages	Key limitations and evidence boundaries	Representative references
Parent-cell programming	3D culture; hypoxic preconditioning; genetic modification of parental ADSCs	May modify EV production, cargo composition, and biological activity before vesicle isolation	Protocol-dependent effects; EV yield does not necessarily predict potency; increased manufacturing complexity	(Han et al., 2018; Shi et al., 2020; Geßner et al., 2021; Li et al., 2025a)
Cargo engineering	Post-isolation co-incubation, electroporation, sonication, and freeze–thaw loading	Enables introduction or enrichment of selected therapeutic cargos	Most methodological evidence derives from the broader EV field rather than ADSC-EVs in DMA; loading procedures may affect EV integrity, cargo stability, and biological activity	(Song et al., 2024)
Surface/targeting engineering	Targeting peptides; aptamers; other membrane-associated ligands	May improve interactions with selected target cells or tissues and enhance delivery specificity	Direct evidence for surface-engineered ADSC-EVs in DMA remains limited; surface modification may alter EV physicochemical properties, biodistribution, and biological activity	(Kang et al., 2020; Fan et al., 2025; Zhao et al., 2025)
Local biomaterial delivery	Hydrogels and other sustained-release matrices, including GelMA-based systems	May improve local retention, protect EVs from premature loss, and prolong therapeutic exposure	EV sources and biomaterial formulations are heterogeneous; biomaterials may contribute to therapeutic effects independently, complicating attribution to EV delivery alone	(Long et al., 2024; Bakhtiari et al., 2025; Cong et al., 2025)
Penetration-enhanced delivery	Dissolving, conductive, or multifunctional microneedle systems	Facilitates localized delivery across superficial tissue barriers and may provide spatially controlled administration	Mainly applicable to locally accessible tissues; multifunctional materials or auxiliary interventions may contribute independently to efficacy; ADSC-EV-specific evidence remains limited	(Xiang et al., 2025; Ye et al., 2025)
Stimuli-responsive delivery	ROS-responsive and other disease-responsive biomaterial systems	May provide greater spatial and temporal control of vesicle or therapeutic cargo release	Current evidence is largely proof-of-concept and frequently involves non-ADSC vesicles or EV-like systems; increasing platform complexity creates additional characterization, reproducibility, and safety challenges	(Ma et al., 2025)

## 5 Organ-specific evidence for ADSC-EVs in diabetic microangiopathy: therapeutic effects and translational limitations

Oxidative stress, inflammation, endothelial dysfunction, and impaired regeneration occur across DMA, but they do not contribute equally to every manifestation. Each affected organ also has its own cellular targets, anatomical barriers, and local disease environment. For this reason, we discuss DR, DKD, DPN, and diabetic chronic wounds separately. For each, we assess the available ADSC-EV evidence and consider its strength, along with the delivery problems and translational limitations that remain.

### 5.1 Diabetic retinopathy: neurovascular protection and evidence limitations

Diabetic retinopathy (DR) is not limited to the retinal vasculature. Endothelial dysfunction, vascular leakage, and capillary nonperfusion are accompanied by neuronal injury, reactive gliosis, and neuroinflammation, indicating that the retinal neurovascular unit is affected more broadly. These changes can develop together and influence one another as the disease progresses. Focusing on pathological angiogenesis alone therefore captures only part of DR, and treatment may need to address several aspects of retinal injury.

Evidence directly linking adipose-derived stem/stromal cell extracellular vesicles (ADSC-EVs) to DR is still sparse. Wu et al. studied retinal microvascular endothelial cells exposed to high glucose and found that exosomes from immortalized adipose-derived mesenchymal stem cells differentiated into pericyte-like cells reduced oxidative stress, apoptosis, permeability, and abnormal angiogenic responses (Wu et al., 2024). The adipose origin makes this study relevant to ADSC-based approaches, but there is an important qualification: the vesicles were collected after pericyte-like differentiation of immortalized ADSCs. They are therefore not equivalent to conventional ADSC-EV preparations.

Other MSC-EV studies help fill some mechanistic gaps but provide a different level of evidence. In an early DR model, MSC-derived exosomes reduced retinal pigment epithelial cell senescence and oxidative stress through PI3K/AKT-Nrf2 signaling and improved retinal structure and function (Bai and Wang, 2024). Because the EVs were not specifically derived from ADSCs, this study supports the broader use of MSC-EVs in DR but cannot be used as direct evidence for ADSC-EVs.

A similar distinction applies to ADSC-EV studies performed outside diabetes. Human ADSC-EVs reduced microglial activation and TLR4/MAPK/NF-κB-associated inflammatory responses in an ocular hypertension model while preserving retinal ganglion cell survival and function (Ji et al., 2024). These results show retinal neuroprotective and anti-inflammatory activity of ADSC-EVs in that model. They do not establish the same effects in DR, where the underlying pathological environment is different.

The studies available so far suggest that EV-based interventions can affect endothelial dysfunction, oxidative stress, neuroinflammation, and neuronal injury. Which of these effects is most relevant may depend on the stage of DR. In earlier disease, preservation of retinal cellular homeostasis and vascular barrier function may be particularly important. Advanced DR, by contrast, also involves ischemia and pathological neovascularization. Angiogenic activity needs particular attention in this context. An effect that promotes repair in an ischemic wound or another diabetic complication may not have the same benefit in the retina, especially in proliferative DR. The consequences of a pro-angiogenic EV effect may therefore depend on both the organ being treated and the stage of disease.

Evidence for ADSC-EVs in DR is still limited to preclinical studies and remains relatively sparse. Studies differ in EV source, isolation and characterization methods, experimental models, dose, route of administration, and retinal outcome measures, making direct comparison difficult. Changes in molecular markers, cellular injury, or vascular permeability also cannot be assumed to reflect lasting recovery of retinal neurovascular function. Delivery remains another unresolved issue because therapeutic effects will depend partly on how EVs are administered and whether they reach the relevant retinal cell populations (Mead and Tomarev, 2020). It is therefore still unclear how reproducible the reported effects are, what dose is appropriate, how responses vary with disease stage, and whether long-term ocular administration is safe.

### 5.2 Restoration of glomerular microenvironmental homeostasis in diabetic kidney disease

Diabetic kidney disease (DKD) is a major microvascular complication of diabetes and a leading cause of end-stage kidney disease. Podocyte injury, mesangial expansion, glomerular basement membrane thickening, chronic inflammation, and renal fibrosis are common pathological features (Kriz et al., 2023). These changes develop within a glomerular environment in which metabolic stress, podocyte dysfunction, endothelial injury, and inflammation are closely linked. Their combined effects progressively impair renal filtration.

Several studies have tested ADSC-EVs directly in experimental DKD. Duan et al. showed that ADSC-EVs containing miR-26a-5p protected podocytes from high-glucose-induced injury by targeting TLR4 and suppressing NF-κB signaling (Duan et al., 2020). Ren et al. reported that ADSC-derived exosomes attenuated diabetic renal injury through the FAM129B/Nrf2/Keap1 axis (Ren et al., 2023). In another study, ADSC-derived exosomal miR-204 reduced oxidative stress and mitochondrial dysfunction by suppressing METTL7A-mediated CIDEC m6A methylation (Jin et al., 2024). These findings directly support effects of ADSC-EVs on several processes relevant to DKD, including podocyte injury, oxidative stress, and mitochondrial dysfunction. The mechanisms identified across studies are different, however, and it is not yet clear whether they occur together or are reproducible across different DKD models.

The heterogeneity of DKD also needs to be considered. Patients differ in clinical phenotype, molecular characteristics, and disease trajectory, and these differences may affect responses to ADSC-EV treatment. Future studies will need to determine whether therapeutic responses vary among DKD phenotypes rather than treating DKD as a uniform disease.

How ADSC-EVs can be delivered efficiently to the kidney is also unresolved. Studies from the broader MSC-EV field have used approaches such as RGD-containing hydrogels to improve EV retention and therapeutic activity in experimental kidney injury (Zhang et al., 2020). These experiments were performed in AKI, not DKD, and cannot be taken as direct evidence for ADSC-EV targeting in diabetic kidney disease. Whether similar approaches can increase renal exposure and improve ADSC-EV efficacy in DKD remains unknown.

Most ADSC-EV evidence in DKD still comes from cell experiments and small-animal models. Studies use different EV preparations, doses, administration routes, disease models, and renal outcome measures. This variability makes the results difficult to compare and limits conclusions about reproducibility. Improvements in podocyte injury, inflammatory signaling, oxidative stress, or mitochondrial function also need to translate into meaningful renal functional outcomes and be confirmed in models that better reproduce chronic human DKD. Until these issues, together with efficient renal delivery, are addressed, optimal dosing, long-term efficacy, and any advantage of ADSC-EVs over other MSC-EV sources remain uncertain.

### 5.3 Diabetic peripheral neuropathy: neurovascular effects and evidence limitations

Diabetic peripheral neuropathy (DPN) is commonly associated with hyperglycemia-induced neural injury, but microvascular dysfunction and reduced neural perfusion also contribute to its pathogenesis (Van Dam et al., 2013; Zhu et al., 2023). Most current treatments focus on glycemic control or symptom relief and have limited ability to reverse established nerve damage. Treating the neural and vascular abnormalities together may therefore be relevant to DPN.

ADSC-EVs have shown effects on both components in preclinical studies. ADSC-derived EVs carrying miR-130a-3p promoted Schwann-cell proliferation and inhibited apoptosis under high-glucose conditions through the DNMT1/NRF2/HIF-1α/ACTA1 axis. The same study also reported increased angiogenesis in a DPN rat model (Chen et al., 2023). Human ADSC-derived exosomes have also shown therapeutic effects in experimental DPN (Cao et al., 2024). These results suggest that ADSC-EVs may act on neural as well as vascular abnormalities. They do not yet show that neurovascular coupling itself is restored. Coordinated recovery of neural and vascular function has rarely been examined and has not been consistently tested in independent models.

Delivery to peripheral nerves is another unresolved issue. The anatomy of peripheral nerves and the blood–nerve barrier can restrict therapeutic access to the endoneurial compartment (Langert and Brey, 2018). Local biomaterial-assisted or targeted delivery could increase EV retention or tissue exposure, but these approaches remain experimental and need to be tested directly with ADSC-EVs in DPN.

Direct evidence for ADSC-EVs in DPN is still relatively sparse, particularly compared with diabetic wound healing and DKD. The available studies use different disease models, EV preparations, doses, and neurological endpoints, and independent replication is limited. Molecular and histological improvements also do not necessarily indicate lasting recovery of nerve conduction, sensory function, or neurovascular coupling. On the current evidence, DPN remains a comparatively early-stage indication for ADSC-EV therapy.

### 5.4 Diabetic chronic wounds: microenvironmental regulation and tissue repair

Diabetic foot ulcers (DFUs) are difficult to heal because several pathological processes persist at the wound site. Prolonged inflammation, microvascular dysfunction, poor oxygen supply, susceptibility to infection, and abnormal extracellular matrix (ECM) remodeling all contribute to wound persistence (Raja et al., 2023). A major difference from acute wounds is that DFUs often remain in an inflammatory state rather than progressing normally through the healing process. Sustained hyperglycemia and inflammatory signaling can maintain pro-inflammatory macrophage activation and interfere with the shift toward reparative phenotypes, further delaying repair (Cai et al., 2023).

Much of the interest in ADSC-EVs for diabetic wounds comes from their reported effects on several of these processes, particularly inflammation, angiogenesis, and tissue repair (Wang et al., 2024). For example, ADSC-derived exosomes carrying circRps5 promoted reparative macrophage polarization through the miR-124-3p axis, with reduced inflammatory responses and improved wound healing (Yin and Shen, 2024). Another study linked the effects of ADSC-EVs on diabetic wound healing to the CCN2/PI3K/AKT pathway (Zhou et al., 2025).

Angiogenesis has been examined in more detail in several experimental studies. ADSC-derived exosomes promoted angiogenesis partly through the miR-146a-5p/JAZF1 axis (Che et al., 2024). When ADSCs were pretreated with hypoxia, their EVs showed enhanced therapeutic activity involving the circ-IGF1R/miR-503-5p/HK2/VEGFA axis (Shi et al., 2024). Hypoxia-preconditioned ADSC-derived exosomes containing circ-Erbb2ip were also reported to promote wound healing through the miR-670-5p/Nrf1 axis, alongside reduced oxidative and inflammatory responses and increased angiogenesis (Tang et al., 2024). These studies implicate different molecular pathways, and it remains unclear how consistently they contribute to ADSC-EV activity across experimental models.

EV retention at the wound site may influence treatment effects. In one study, low-intensity pulsed ultrasound increased ADSC-Exo uptake and kept the vesicles in diabetic wounds for longer, alongside increased angiogenesis and wound repair (Zhong et al., 2024). Whether this method can be standardized and whether it consistently performs better than unmodified ADSC-EV administration are still unclear.

Diabetic wounds have been studied more extensively with ADSC-EVs than several of the other DMA manifestations covered in this review. Even so, the studies differ considerably in wound models, EV isolation and characterization, dose, route of administration, and outcome measures. This makes their results difficult to compare directly. There is also a substantial gap between wound closure in rodents and durable healing of human DFUs. Human wounds are further complicated by infection, ischemia, neuropathy, and differences among patients. The preclinical findings are encouraging, but they cannot yet establish clinical efficacy. Studies in standardized large-animal models, together with outcome measures that better reflect human wound healing, are needed to determine how well these results translate to DFUs.

### 5.5 Multitarget effects and the potential for pathological network remodeling by ADSC-EVs

The evidence reviewed above shows that ADSC-EV therapy cannot be considered in the same way across all DMA manifestations. DR, DKD, DPN, and diabetic wounds differ in anatomy, local microenvironment, and barriers to delivery, as summarized in Figure 4. Yet several pathological processes recur across these complications, including oxidative stress, inflammation, endothelial dysfunction, mitochondrial abnormalities, and impaired regeneration (Chen et al., 2025). Their relative importance differs among organs, but this overlap makes interventions that affect more than one process relevant to DMA.

**FIGURE 4:**
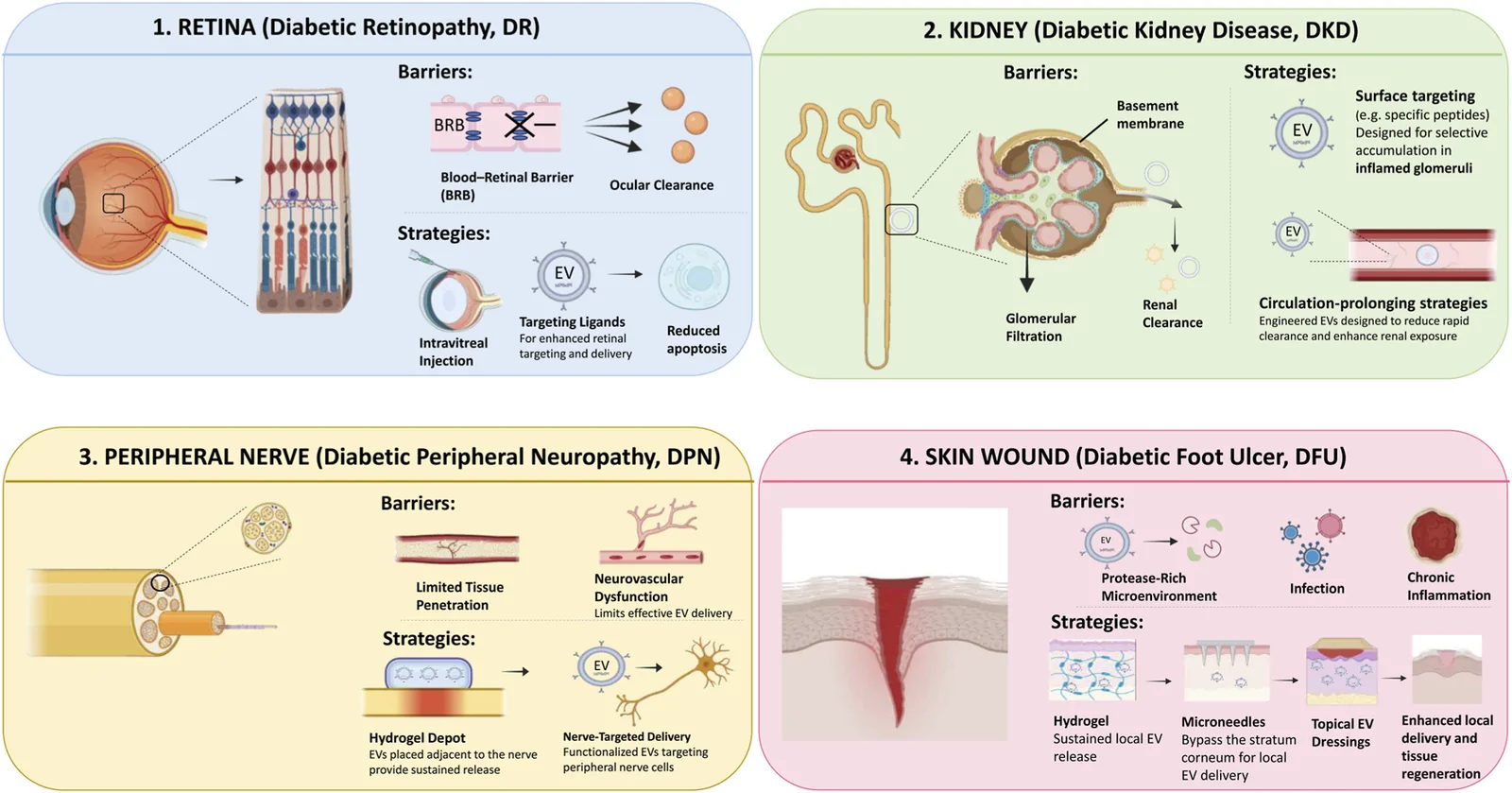
Organ-specific delivery strategies for engineered ADSC-EVs in diabetic microangiopathy. ADSC-EV therapy across major diabetic microvascular complications requires organ-specific delivery strategies: (1) DR—intravitreal injection and ligand-based targeting to overcome the BRB and intraocular clearance; (2) DKD—surface-functionalized EVs and circulation-prolonging strategies to enhance renal delivery while limiting systemic clearance; (3) DPN—hydrogel depots or nerve-targeted functionalization to improve local retention and delivery to peripheral nerves; and (4) DFU—hydrogels, microneedles, or topical dressings to protect EVs within the damaged, infected, and inflamed wound microenvironment and enhance local retention and tissue repair. Created in BioRender. Zhu, X. (2026) https://BioRender.com/fq4j645.

ADSC-EVs may provide such multitarget activity. Like EVs from other sources, they can carry regulatory RNAs, proteins, lipids, and other bioactive molecules (Liu et al., 2023). These components may act on different biological processes within the same disease setting. We do not, however, regard this alone as evidence of pathological network remodeling. An EV preparation can affect several pathways without changing the interactions that sustain the disease. Multiple cargos and multiple biological effects therefore support multitarget activity, but network remodeling requires a different level of evidence.

This distinction is apparent across the organ-specific studies. In retinal models relevant to DR, adipose-derived or ADSC-derived EV preparations have been linked to changes in endothelial injury, neuroinflammation, and neuronal damage, although direct ADSC-EV evidence in diabetic retinopathy remains limited. DKD studies report effects on podocyte injury, oxidative stress, and mitochondrial dysfunction. In DPN, ADSC-EVs may support peripheral nerve repair, but whether neural and vascular function recover in a coordinated manner remains unclear. Diabetic wound studies cover a wider range of responses, including macrophage polarization, angiogenesis, oxidative and inflammatory responses, and tissue repair. These findings show that ADSC-EVs can affect different parts of the disease process. They do not yet show that these effects are causally connected or that disease-sustaining interactions have been durably altered.

Exosome-mediated cell–cell communication offers a possible route through which interconnected pathological processes could be influenced (Chen et al., 2026). On the available evidence, however, we consider pathological network remodeling a working hypothesis rather than a demonstrated mechanism of ADSC-EVs. Most studies still focus on individual cargos, pathways, or phenotypic outcomes. Few examine whether changes in one pathological process alter another or whether the relationships among these processes change over time.

A key question is whether the effects of ADSC-EVs on different pathological processes are actually connected. Addressing this will require more than measuring individual pathways or endpoints. Single-cell sequencing, spatial transcriptomics, multi-omics profiling, and network-based computational analysis can provide information across cell types and molecular pathways. Together with longitudinal functional assessment and causal perturbation, these methods may help distinguish changes in the relationships among pathological processes from several responses occurring in parallel. They may also help identify which disease states are more likely to respond to particular ADSC-EV effects and support more individualized EV-based therapeutic strategies.

## 6 Precision translation of ADSC-EVs: challenges and future perspectives

Clinical studies of EV-based therapies are increasing, but ADSC-EV research is still dominated by preclinical work. Rodent models can reveal mechanisms and treatment effects, but they capture only part of the clinical complexity of long-standing diabetes. Patients may have persistent metabolic abnormalities alongside atherosclerosis and chronic inflammation and may be taking several medications at the same time. These features are difficult to reproduce together in experimental models.

There is also considerable variation among patients with diabetic microangiopathy. Metabolic status, immune microenvironment, disease stage, and comorbidities differ, and these differences may affect treatment response. The same EV formulation may therefore not work equally well for every patient or at every stage of disease. EV cargo and delivery may also need to reflect the underlying pathological features. Biomarkers, multi-omics, and patient stratification could eventually help with these decisions, but this type of precision EV therapy remains largely conceptual and requires prospective validation.

Before these more individualized approaches can be considered clinically, several basic problems with ADSC-EV development remain. Products need to be manufactured consistently, and their biodistribution, delivery, and safety need to be defined more clearly. Regulatory evaluation will also depend on how the EVs are produced and modified. These issues affect EV therapeutics more broadly, but donor characteristics, culture conditions, and engineering modifications can introduce further variability into ADSC-EV products. A modification that improves therapeutic activity is useful only if the resulting product can also be produced reproducibly, characterized reliably, evaluated for safety, and used feasibly in clinical practice.

### 6.1 Industrial standardization and drug development of EV therapeutics

Turning EVs into reproducible therapeutic products requires more than producing them at high yield. Unlike conventional drugs made under tightly defined chemical processes, EVs are biological products whose properties can change with the donor, the state of the parental cells, culture conditions, and the method used for isolation (Thakur and Rai, 2024). A GMP-compliant process therefore needs to control these sources of variation while maintaining consistent biological activity.

The Minimal Information for Studies of Extracellular Vesicles (MISEV) guidelines developed by the International Society for Extracellular Vesicles (ISEV) address many of the methodological issues that affect EV research. They provide recommendations for EV separation and characterization, molecular markers, functional assessment, and evaluation of potential contaminants (Welsh et al., 2024; Zhang et al., 2024). Their purpose, however, is different from that of GMP. MISEV improves how EV studies are performed and reported, whereas GMP requirements govern how a clinical-grade therapeutic product is manufactured and released.

Isolation and purification are important sources of variation in the final EV preparation. Ultracentrifugation, size-exclusion chromatography, tangential-flow filtration, and polymer precipitation do not recover identical products and can differ in EV yield, purity, and cargo retention (Dong et al., 2020; Takakura et al., 2024). Differences attributed to EV biology may therefore sometimes also reflect how the vesicles were isolated and processed.

Some of the variability in ADSC-EV products can be traced back to the parental cells. Donor age and cell expansion can alter the miRNA profiles of sEVs derived from human ADSCs (Tsubaki et al., 2025). Cell passage and culture conditions may also affect the parental-cell state, while differences among donors add another source of variation (Loria et al., 2024). These factors can ultimately influence the EVs that are produced and need to be controlled to maintain consistency between manufacturing batches.

This means that quality control cannot begin only after the EVs have been isolated. It needs to cover donor selection, cell expansion, EV production and purification, and final product release. Particle concentration, size distribution, identity-associated markers, purity, sterility, endotoxin levels, and stability can be used to characterize different aspects of the product (Takakura et al., 2024; Thakur and Rai, 2024). None of these measurements, however, shows on its own whether the EV preparation has the intended therapeutic activity. Functional potency assays relevant to the intended indication are needed for that purpose.

Particle number is therefore only one measure of an ADSC-EV product. Producing a large number of particles has limited value if their characteristics or biological activity vary from batch to batch. For clinical development, the manufacturing process needs to generate EV preparations with consistent identity, purity, potency, stability, and safety.

### 6.2 Biodistribution heterogeneity and precision delivery

One difficulty with systemic EV therapy is that much of the administered dose may never reach the intended tissue. After intravenous administration, EVs are often cleared by the mononuclear phagocyte system (MPS) and accumulate in the liver, spleen, and lungs (Driedonks et al., 2022; Chung et al., 2024; Rosenkrans et al., 2024). This can substantially reduce the amount available to other target tissues (Kang et al., 2021). Similar patterns have been reported for EVs from different cellular sources, so they should not be regarded as specific to ADSC-EVs.

Important aspects of EV pharmacokinetics are still poorly defined. It remains unclear how long EVs circulate, how they are distributed among tissues, and how much these patterns change with the route of administration (Kang et al., 2021; Driedonks et al., 2022). The diabetic environment may introduce further variation. Vascular dysfunction, chronic inflammation, and endothelial injury could affect EV transport, cellular uptake, or therapeutic responses, but their effects on ADSC-EV pharmacokinetics and biodistribution have not been well characterized.

The practical delivery problem is different in each DMA manifestation. The blood–retinal barrier may restrict EV access to relevant retinal cells in DR (Zhu et al., 2024). In DKD, obtaining sufficient EV exposure in the kidney while maintaining tissue selectivity remains difficult (Tang et al., 2022). Peripheral nerves are widely distributed and relatively inaccessible, which complicates delivery in DPN (Langert and Brey, 2018). Diabetic wounds are easier to reach directly, but local administration does not eliminate problems such as EV loss and degradation. These differences argue against using the same delivery approach across all DMA manifestations.

The choice of cargo therefore cannot be separated from the question of how the EVs will reach the target tissue. Ligand-mediated targeting, hydrogel-assisted retention, microneedle-based delivery, magnetic guidance, and ROS- or pH-responsive systems have all been investigated for this purpose. Much of this work, however, comes from EVs other than ADSC-EVs, and direct evidence across DMA indications remains limited. Local delivery is more feasible for diabetic wounds, whereas reliable delivery to the retina, kidney, and peripheral nerves remains difficult. Quantitative *in vivo* imaging, together with pharmacokinetic and biodistribution analysis, may help show where administered EVs actually go and which delivery methods are better suited to each DMA manifestation.

### 6.3 Safety considerations and long-term risk assessment

ADSC-EVs contain no viable cells, which may avoid some concerns associated with cell transplantation, such as uncontrolled cell proliferation. Their long-term safety is much less clear. Clinical studies of EV-based therapies have generally reported acceptable short-term tolerability, but they have involved relatively few patients and provide limited long-term follow-up (Van Delen et al., 2024). For DMA, this is an important gap because a chronic, progressive disease may require repeated treatment. The consequences of repeated EV exposure are not yet well understood (Takakura et al., 2024).

Not all potential risks would be detected by conventional acute toxicity testing. EVs can alter immune responses and may also act in tissues other than the intended target. Donor-cell characteristics and manufacturing procedures can introduce further sources of risk (Claridge et al., 2021). Angiogenic activity is another consideration because its effects depend on the disease context. Promoting angiogenesis may be useful in some settings but undesirable in others if the response is inappropriate or excessive, although direct evidence for this risk remains limited. There is also little information on possible interactions between EV products and medications commonly used by patients with diabetes.

The biological effects of EVs may also persist beyond the period captured by short-term studies. Because they can influence several pathways and alter local tissue environments, longer follow-up is needed to examine possible changes in immune function, tissue remodeling, and tissue homeostasis.

Safety may also depend on who receives the treatment. Patients with diabetes often have chronic inflammation, vascular dysfunction, metabolic abnormalities, and other comorbidities. These features are difficult to reproduce in healthy animals or simplified disease models and may change the response to EV administration. Patients may also be taking several medications, another factor that is rarely represented in preclinical studies.

For these reasons, long-term assessment of ADSC-EVs needs to capture delayed effects as well as immunogenicity, biodistribution, and adverse events. The product itself also needs to be defined consistently. Without reproducible quality and potency standards, safety results from different studies are difficult to compare and may not describe equivalent EV products (Takakura et al., 2024).

### 6.4 Regulatory classification and approval pathways

There is currently no single regulatory pathway for EV therapeutics across different jurisdictions. How a product is classified depends on what the product is, how extensively it has been modified, its intended use, and the regulatory framework in which it is developed (Stawarska et al., 2024; Limongi et al., 2026). In Europe, recent guidance indicates that non-substantially modified EVs are outside the current definition of advanced therapy medicinal products (ATMPs). Other jurisdictions may regulate therapeutic EVs under existing drug or biological-product frameworks (Limongi et al., 2026).

These differences affect how EV products are developed and evaluated. More consistent approaches to classification and to the assessment of product quality and safety will require coordination among regulators, researchers, and industry. The level of engineering is also relevant. An EV product with a relatively simple manufacturing process and a clearly defined indication is likely to be easier to characterize and compare across batches than a highly engineered multifunctional product. Additional modifications can make it harder to establish comparability and can add new questions for characterization, safety assessment, and regulatory review (Limongi et al., 2026).

For ADSC-EVs, much of the regulatory challenge comes down to whether the product can be defined and produced consistently. Different batches need to show comparable characteristics and biological activity, which requires reproducible manufacturing, appropriate potency assays, clearly specified quality attributes, and transparent risk assessment. Without these elements, it becomes difficult to establish that different batches represent the same therapeutic product or to evaluate them consistently in clinical development.

### 6.5 Translational priorities across different forms of diabetic microangiopathy

The path to clinical testing is not the same for every form of diabetic microangiopathy. Diabetic wounds may be easier to approach initially because treatment can be applied directly to the affected tissue and outcomes such as wound closure can be followed over time. Preclinical systematic reviews and meta-analyses have reported beneficial effects of EV-based interventions in diabetic wound models, including studies of ADSC-derived EVs (Soltani et al., 2024). Work with BM-MSC-derived EVs and miRNA-engineered exosomes also supports the broader use of EV-based approaches in diabetic wound healing, but these studies do not provide direct evidence for ADSC-EVs (Du et al., 2024; Jian et al., 2024).

DR, DKD, and DPN pose different problems. In DR, ocular barriers and the choice of administration route affect access to the relevant retinal cells (Zhu et al., 2024). For DKD, the challenge is to obtain sufficient EV exposure in the kidney while maintaining tissue selectivity (Tang et al., 2022). Peripheral nerves are distributed across multiple anatomical sites and are relatively difficult to access, making both targeted delivery and functional repair more difficult in DPN (Langert and Brey, 2018).

These practical differences should influence which indications are taken forward first. A strong biological effect in an experimental model does not by itself make an indication ready for clinical translation. It also matters whether the target tissue can be reached reliably, whether local administration is possible, and whether treatment effects can be measured using clinically meaningful endpoints. By these criteria, diabetic wounds may currently provide a more practical setting for early ADSC-EV translation than DR, DKD, or DPN. This comparison does not imply that ADSC-EVs are clinically more effective in diabetic wounds. It reflects the current preclinical evidence together with differences in tissue accessibility and delivery feasibility. Table 3 compares the main translational opportunities, remaining challenges, and current evidence maturity across the four DMA manifestations.

**TABLE 3:** Translational opportunities, challenges, and current evidence maturity of ADSC-EVs across different manifestations of diabetic microangiopathy.

Disease	Major translational advantages	Key challenges	Clinical maturity	References
Diabetic retinopathy (DR)	Well-defined target organ; local ocular administration is feasible	Ocular barriers and route-dependent retinal exposure; long-term ocular safety	Preclinical; limited ADSC-EV-specific evidence	(Zhu et al., 2024)
Diabetic kidney disease (DKD)	High unmet clinical need; multiple experimentally supported renal targets	Limited renal targeting efficiency; chronic dosing and functional validation	Preclinical	(Duan et al., 2020; Ren et al., 2023; Jin et al., 2024)
Diabetic peripheral neuropathy (DPN)	Potential to address neural injury and associated microvascular dysfunction	Anatomically dispersed/inaccessible peripheral nerves; functional nerve recovery difficult to establish	Preclinical; comparatively limited ADSC-EV evidence	(Langert and Brey, 2018; Chen et al., 2023; Cao et al., 2024)
Diabetic foot ulcer (DFU)	Local administration feasible; wound-healing endpoints directly measurable	Infection, ischemia, neuropathy, patient heterogeneity, and local EV retention	Relatively advanced preclinical evidence; clinical translation remains limited	(Soltani et al., 2024; Yin and Shen, 2024; Zhong et al., 2024)

### 6.6 Early clinical translation of EV therapeutics

Clinical testing of EV-based therapies for diabetic complications is still limited. Most of the evidence discussed in this review comes from preclinical models, and experimental regenerative or protective effects cannot be assumed to translate into clinical benefit. EV-associated mechanisms have been implicated in diabetic wound repair, for example, but mechanistic evidence of this kind is different from direct clinical evidence that an administered EV product is effective (Xie et al., 2024).

A few registered studies show that EV-based products are beginning to be tested in patients. A completed pilot study used human adipose tissue-derived exosomes/EVs together with a hydrogel for wound treatment (NCT05475418). Human adipose tissue-derived EVs combined with hyaluronic acid have also been investigated in a randomized controlled multicenter study of recalcitrant wounds (NCT06253975). These studies are relevant to adipose-derived EV therapy, but the products were obtained directly from adipose tissue rather than from cultured ADSCs. They should therefore not be treated as clinical studies of ADSC-EVs. In diabetic foot ulcers, another clinical study has investigated human umbilical cord mesenchymal stromal cell derivatives containing EVs/exosomes (NCT06825884). Even at this early stage, the products entering clinical studies differ in their source and definition.

This distinction is important when judging how far ADSC-EVs have progressed clinically. The studies above show that different EV-based products have reached clinical investigation, but they do not establish clinical efficacy for cultured ADSC-derived EVs in diabetic microangiopathy. Results obtained with adipose tissue-derived EVs, UC-MSC-derived products, or other EV preparations cannot simply be applied to ADSC-EVs because the source, manufacturing process, purification, characterization, and formulation may all influence the final product and its therapeutic performance. Table 4 summarizes representative registered clinical studies relevant to EV-based therapies for diabetic microangiopathy. For cultured ADSC-EVs, further clinical development will depend on clearly defined products, standardized manufacturing and characterization, appropriate dosing and administration, and prospective assessment of safety and clinically meaningful efficacy endpoints.

**TABLE 4:** Representative registered clinical studies relevant to translating EV-based therapies for diabetic microangiopathy.

EV source	Indication	Route of administration	Study status	ClinicalTrials.gov identifier
Human adipose tissue-derived exosomes/EVs	Wounds and injuries	Local hydrogel-based delivery	Completed	NCT05475418
Human adipose tissue-derived EVs	Recalcitrant full-thickness wounds	Local EV delivery combined with hyaluronic acid	Recruiting	NCT06253975
Human umbilical cord mesenchymal stromal cell derivatives, including EVs/exosomes	Diabetic foot ulcers	Perilesional injection	Active, not recruiting; clinical results published	NCT06825884

The listed studies involve heterogeneous EV, sources. Adipose tissue-derived EVs, are not equivalent to EVs, specifically derived from cultured ADSCs, and the study involving umbilical cord MSC, derivatives represents broader MSC-EV, clinical evidence rather than ADSC-EV-specific clinical evidence.

### 6.7 Toward a personalized precision EV therapeutic platform

Patients with DMA differ in metabolic status, inflammatory and immune profiles, disease stage, and comorbidities. These differences may also affect how they respond to EV-based treatment. It is therefore unlikely that one EV formulation will be equally suitable for every patient or across all DMA manifestations. A more individualized approach would take the disease phenotype and treatment setting into account when deciding how an EV product should be used or designed.

New profiling methods may help define these differences more clearly. Single-cell analysis, spatial transcriptomics, multi-omics profiling, and computational modeling can be used to examine molecular variation across patients and disease states and may identify features associated with treatment response. In the future, such information could help stratify patients or guide the selection and engineering of EV products. For ADSC-EVs, however, matching a particular cargo or formulation to a patient’s disease phenotype remains a prospective idea and has not been clinically validated (Cheng, 2024).

Computational and AI-assisted methods could also help bring together data on EV cargo, delivery, manufacturing, and therapeutic response. At present, their use in ADSC-EV development is largely exploratory. Any predictions or design choices generated through these methods will still need to be tested experimentally before their clinical value can be determined.

A precision approach to ADSC-EV therapy will therefore depend on more than identifying patient subgroups or designing different EV formulations. The products themselves must still be manufactured reproducibly, assessed with standardized potency assays, delivered reliably, and evaluated for long-term safety. For now, personalized EV therapy is a framework for future development rather than an established clinical strategy.

## 7 Conclusions and future perspectives

Diabetic microangiopathy is shaped by several pathological processes that develop together and influence one another. Oxidative stress, inflammation, immune dysregulation, endothelial dysfunction, metabolic and mitochondrial abnormalities, fibrosis, and impaired regeneration are found across DMA, but their contribution is not the same in every affected organ. This matters therapeutically. Once these processes become linked, correcting one abnormal pathway may leave much of the disease process unchanged.

ADSC-EVs are relevant in this setting because they can carry multiple bioactive components and act on more than one disease-related process. This should not be mistaken for a property unique to ADSC-EVs. Other EV populations share many of the same biological features, including multimolecular cargo delivery and the capacity for engineering. The literature reviewed here supports ADSC-EVs as a promising therapeutic candidate, but it does not show that they are generally superior to EVs from other MSC sources. The evidence is also uneven. Diabetic wounds have been studied more extensively, whereas direct ADSC-EV evidence in DR and DPN is much thinner, and clinical evidence for EVs produced from cultured ADSCs is still scarce.

Engineering can improve cargo loading, targeting, retention, or release, but every added modification brings its own trade-offs. A more sophisticated EV product may perform better experimentally while becoming harder to manufacture, characterize, track *in vivo*, and evaluate for safety. The best design is also unlikely to be the same for every DMA manifestation. Local retention is particularly relevant to diabetic wounds, whereas the retina, kidney, and peripheral nerves present very different delivery barriers. The engineering strategy should follow the biological and anatomical problem rather than the other way around.

The next step for ADSC-EV research is not simply to produce more positive preclinical results. The field needs to establish whether the same EV product can be manufactured consistently, how its potency should be measured, where it goes after administration, how much reaches the intended tissue, and what happens after repeated exposure. Just as importantly, conclusions about ADSC-EVs should come from ADSC-EV studies whenever possible. Findings from other MSC-EV sources are valuable for defining mechanisms and guiding experimental design, but they cannot substitute for source-specific evidence.

Multi-omics, spatial profiling, quantitative biodistribution analysis, and computational approaches can provide a more detailed view of how EV treatment affects diseased tissues, while advances in bioengineering and GMP-compatible production may improve product design and manufacturing. These tools will be most informative when paired with clinically relevant models, functional endpoints, and clearly defined EV preparations. For now, pathological network remodeling and precision EV therapy are useful frameworks for asking new questions, not established explanations for how ADSC-EVs work. The more immediate question is whether their reported multitarget effects can be reproduced and converted into meaningful functional benefits in the different forms of diabetic microangiopathy.

## Glossary

AGE/RAGE: Advanced glycation end products/receptor for advanced glycation end products
ADSCs: Adipose-derived stem cells
ADSC-EVs: Adipose-derived stem cell-derived extracellular vesicles
AI: Artificial intelligence
ATMPs: Advanced therapy medicinal products
BDNF: Brain-derived neurotrophic factor
BRB: Blood–retinal barrier
CAT: Catalase
circRNAs: Circular RNAs
DFU: Diabetic foot ulcer
DKD: Diabetic kidney disease
DMA: Diabetic microangiopathy
DPN: Diabetic peripheral neuropathy
DR: Diabetic retinopathy
ECM: Extracellular matrix
eNOS: Endothelial nitric oxide synthase
EVs: Extracellular vesicles
GelMA: Gelatin methacryloyl
GMP: Good manufacturing practice
GPx: Glutathione peroxidase
HO-1: Heme oxygenase-1
ICAM-1: Intercellular adhesion molecule-1
IL-1β: Interleukin-1 beta
IL-6: Interleukin-6
ISEV: International Society for Extracellular Vesicles
lncRNAs: Long non-coding RNAs
miRNAs: MicroRNAs
MISEV: Minimal Information for Studies of Extracellular Vesicles
MMPs: Matrix metalloproteinases
MPS: Mononuclear phagocyte system
MSCs: Mesenchymal stem cells
NF-κB: Nuclear factor-kappa B
NGF: Nerve growth factor
Nrf2: Nuclear factor erythroid 2-related factor 2
NVU: Neurovascular unit
PI3K/Akt: Phosphoinositide 3-kinase/protein kinase B
PKC: Protein kinase C
RAS: Renin–angiotensin system
ROS: Reactive oxygen species
scRNA-seq: Single-cell RNA sequencing
SOD: Superoxide dismutase
ST: Spatial transcriptomics
STZ: Streptozotocin
TGF-β: Transforming growth factor-beta
TIMPs: Tissue inhibitors of metalloproteinases
TNF-α: Tumor necrosis factor-alpha
VCAM-1: Vascular cell adhesion molecule-1
VEGF: Vascular endothelial growth factor
